# The effect of neighborhood walkability on changes in physical activity and sedentary behavior during a 12-week pedometer-facilitated intervention

**DOI:** 10.1371/journal.pone.0278596

**Published:** 2022-12-01

**Authors:** Gavin R. McCormack, John C. Spence, Tara-Leigh McHugh, W. Kerry Mummery

**Affiliations:** 1 Cumming School of Medicine, University of Calgary, Calgary, Alberta, Canada; 2 Faculty of Kinesiology, University of Calgary, Calgary, Alberta, Canada; 3 School of Architecture, Planning and Landscape, University of Calgary, Calgary, Alberta, Canada; 4 Faculty of Sport Sciences, Waseda University, Tokyo, Japan; 5 Faculty of Kinesiology, Sport, and Recreation, University of Alberta, Edmonton, Alberta, Canada; Tabriz University of Medical Sciences, ISLAMIC REPUBLIC OF IRAN

## Abstract

**Background:**

Pedometer-facilitated interventions encourage physical activity via the accumulation of steps. Mixed evidence suggests that neighborhood walkability might influence the effectiveness of physical activity interventions, including pedometer-facilitated interventions. Our study investigated the moderating effect of neighborhood walkability on immediate (4-week) and short-term (12-week) changes in self-reported neighborhood-specific leisure and transportation walking, leisure-based moderate and vigorous-intensity physical activity, and leisure-based screen time during a pedometer-facilitated intervention (UWALK).

**Methods:**

This quasi-experiment undertaken in Calgary (Canada) compared behavior changes during the 12-week intervention between two neighborhood groups classified as ‘walkable’ or ‘car dependent’ based on Walk Score®. Of the 573 volunteers (adults in the contemplation and preparation stages of physical activity behavior change), 466 participated in UWALK. Surveys captured sociodemographic characteristics, perceived neighborhood walkability, neighborhood preferences, motivation, physical activity and screen-based leisure. Covariate-adjusted linear mixed models estimated the differences in physical activity and leisure screen time between the neighborhood walkability groups at baseline, 4-weeks, and 12-weeks.

**Results:**

UWALK participants included mostly females (83%) and had an average age of 49.2 years. Weekly minutes of walking for transport inside the neighborhood was higher (p < .001) among participants from walkable versus car dependent neighborhoods at baseline (42.5 vs. 21.1), 4-weeks (81.2 vs. 48.2), and 12-weeks (87.2 vs. 48.0). Regardless of neighborhood walkability, all physical activity outcomes were higher and leisure screen time lower at 4-weeks and 12-weeks compared with baseline. We found no significant neighborhood group by time interactions.

**Conclusions:**

Pedometer-facilitated interventions may be effective for supporting short-term changes in physical activity and sedentary behavior even among adults residing in low walkable neighborhoods.

## Introduction

Regular participation in physical activity can improve cardiovascular, metabolic, and mental health [1]. Even small increases in physical activity, especially among inactive adults can provide health benefits [2]. Interventions that increase physical activity are therefore important for health promotion. While different types of physical activity intervention exist [3,4], pedometer-facilitated interventions can be low cost and broad reaching and support increases in low-to-moderate intensity physical activity by encouraging adults to walk more [5]. Pedometer-facilitated interventions are effective at increasing physical activity [3,5,6] and improving health (e.g., weight loss) [7].

Pedometer-facilitated interventions typically do not require that participants adhere to a prescribed physical activity routine or to use certain facilities or equipment although they might encourage participants to achieve a step goal (e.g., 10,000 steps/day) [8–10]. Thus, participants in pedometer-facilitated interventions accumulate their physical activity of their own accord meaning they can undertake their preferred physical activities how and where they choose. Because pedometer-facilitated interventions encourage participants to accumulate steps, walking is a common physical activity undertaking during this type of intervention, especially among inactive adults [11,12].

There are several commonly reported barriers to physical activity [13–15]. Notably, the neighborhood built environment can support or constrain physical activity. For instance, neighborhood built characteristics, such as walkability, are associated with physical activity including walking for different purposes [16–19]. Neighborhoods that offer higher pedestrian connectivity, a mix of land use or destinations, recreational opportunities such as parks, and higher levels of personal and traffic safety support physical activity [16,17,19,20]. Neighborhoods with higher walkability may also encourage residents to replace short-car trips with active transport modes such as walking and cycling [21,22].

Neighborhood walkability is often estimated using Walk Score® —a publically available measure that reflects the proximity to community amenities (e.g., grocery stores, coffee shops, parks, stores) [23]. Walk Score® is positively associated with transportation and leisure walking and physical activity [21,24–30] including when Walk Score® is categorized [28,31]. For example, Hajna et al. [29] found a two-fold increase in utilitarian walking for at least one-hour per week among adults residing in neighborhoods in the highest versus lowest quartile of Walk Score® but observed no significant difference in accelerometer-measured steps. Using predefined Walk Score® classifications (i.e., very car dependent, car dependent, somewhat walkable, very walkable, and walker’s paradise), Twardzik et al. [28] found lower minutes of accelerometer-measured moderate-to-vigorous intensity physical activity among adults residing in less walkable neighborhoods versus those residing in neighborhoods classified as very walkable/walker’s paradise. In contrast, the researchers also found more minutes of total physical activity (light, moderate, and vigorous intensity) among adults residing in very car-dependent versus very walkable/walker’s paradise neighborhoods controlling for individual and neighborhood level socioeconomic status. Notably, these studies did not differentiate between the contexts in which physical activity was undertaken (e.g., inside versus outside the neighborhood) and only included a limited range of physical activity outcomes.

A recent systematic review of 20 experimental studies found mixed evidence of the objectively-measured neighborhood built environment affecting the accumulation of physical activity during individual-level interventions [32]. Seventy percent of the studies reviewed found at least one neighborhood built environment variable that either positively or negatively impacted the effectiveness of a physical activity intervention [32]. For instance, during a 12-month pedometer-facilitated intervention implemented in Scotland, objectively measured neighborhood built features related to dangerous and busy roads and traffic signals and pedestrian signage were negatively associated with steps while features related to commercial and residential land use were positively associated with steps [33]. Further, Hino et al. [34] found that neighborhood bus stop density was positively associated with self-reported steps, while Hino et al. [35] found proximity to the nearest railway station and higher population density were positively associated with recorded steps among adults exposed to a city-wide pedometer-facilitate intervention in Japan.

In a Canadian sample, we previously estimated associations which included perceived and objectively measured walkability (i.e., Walk Score®), but found that only perceived walkability was positively associated with the accumulation of daily steps during a 12-week pedometer-facilitated intervention (UWALK) [36]. In follow-up interviews, participants identified proximity to destinations and recreation facilities, aesthetics, nature, and pathways as physical activity enablers and unstimulating environments, garbage, homeless people, off-leash dogs, and the lack of destinations and green space as physical activity barriers during UWALK [37]. UWALK participants were also more of aware of their patterns of sedentary and physical activity, and they felt encouraged to do more leisure and transportation walking in and around their neighborhoods [37].

Pedometers encourage and can capture the accumulation of physical activity, however, these devices do not collect information about the contexts in which physical activity is accumulated, nor do they provide information about the purpose for which physical activity was undertaken. In this study we extend our previous analysis of UWALK [36,37] by examining the moderating effect of neighborhood walkability (Walk Score®) on immediate (4-week) and short-term (12-week) changes in self-reported neighborhood-specific leisure and transportation walking, leisure-based moderate and vigorous-intensity physical activity, and leisure-based screen activity during the pedometer-facilitated intervention. We hypothesized that changes in self-reported physical activity at 4 weeks and 12 weeks during the intervention would be significantly higher for adults residing in neighborhoods that were more versus less walkable.

## Materials and methods

### Sample recruitment

The study design and recruitment has been described elsewhere [38]. Briefly, between May 2016 and August 2017, adult volunteers were recruited to participate in an internet-based pedometer intervention via newsletters, websites, and social media (i.e., Facebook and Twitter) of 147 community associations, twitter tweets to members of the University of Calgary, City of Calgary, and Federation of Calgary Communities, and via an advertisement in a freely available local newspaper (Metro News). Interested participants contacted the research coordinator and were screened for eligibility. To qualify, participants were at least 18 years of age, in the “contemplation” or “preparation” stages of physical activity behavior change, not previously or currently registered in UWALK, reported no mobility constraints, and had internet access. Participants were identified as physical activity contemplators if they reported “true” to the following two statements: “*I currently do not participate in recreational or transportation-related physical activity*” and “*I intend to participate in recreational or transportation-related physical activity in the next 3 months*”. Participants were identified as physical activity preparers if they reported “false” to the following two statements: “*I currently do not participate in recreational or transportation-related physical activity*” and “*I am currently participating in recreational or transportation-related physical activity ≥3 days/week*”. Thus participants were considered to have low levels of physical activity [39]. Only one adult per household was eligible to participate in the study.

### Procedures

This quasi-experiment compared behavior at baseline and during UWALK (4 weeks and 12 weeks) between two neighborhood groups classified as ‘walkable’ or ‘car dependent’ based on pre-determined Walk Score® cut-points. Studies examining the influence of the built environment on intervention-facilitated physical activity have typically focused on interventions implemented for 6-months or longer with few studies examining interventions of shorter length [32]. Eligible participants (n = 573) completed a baseline telephone survey capturing sociodemographic characteristics, perceived neighborhood walkability, neighborhood preferences, physical activity motivation (i.e., self-efficacy and intention), physical activity and screen-based leisure. After completing the survey, participants were mailed instructions on how to use and wear the included pedometer, how to register and track physical activity on the UWALK website, a daily tracking sheet, and UWALK promotional material [38]. The study involved a 12-week internet-delivered pedometer intervention during which participants recorded their steps daily or weekly into a website to track their own progress (www.uwalk.ca) and completed two follow-up online surveys (i.e., at 4-weeks and 12 weeks after the baseline survey). Physical activity and screen-based leisure were re-measured during each of the follow-up surveys. UWALK was informed by previous community-based, theory-driven, pedometer-based interventions [8–10]. It used established health promotion approaches based on social cognitive theory to encourage individuals to walk as a means of increasing their physical activity, including achieving 10,000 steps per day [40]. Participants were encouraged to use activity trackers (e.g., pedometers) to self-monitor their physical activity. For our study, participants received a Piezo StepX pedometer [41]. They were instructed to wear the pedometer on their hip at all times except while sleeping, swimming, bathing, or undertaking contact sports.

All procedures performed in studies involving human participants were in accordance with the ethical standards of the University of Calgary Conjoint Health Research Ethics Board (REB15-2944) and the University of Alberta Human Research Ethics Board (Pro00110373) with the 1964 Helsinki declaration and its later amendments or comparable ethical standards. All participants provided oral consent prior to their involvement in the study.

### Measures

#### Objectively measured neighborhood walkability

Neighborhood walkability was estimated using Walk Score®; a publicly available walkability index that reflects proximity to nearby walkable destinations (i.e., grocery stores, coffee shops, restaurants, bars, movie theatres, schools, parks, libraries, book stores, fitness centres, drug stores, hardware stores, clothing/music stores) [23,42]. Walk Score® values range from 0 to 100 with higher scores reflecting higher walkability. Complete household address information was not available for participants, thus each participant’s residential 6-digit postal code was assigned a Walk Score® (extracted from www.walkscore.com in 2017) which was used to categorize neighborhoods as “car dependent” (value <50) or “walkable” (value 50–100)” [23]. In Calgary (Canada), postal code locations are considered an accurate surrogate measure of the geographical location of households, with households located within mean and median Euclidean distances of 146.2 (SD 369.3) meters and 69.1 meters, respectively, of the true address location [43].

#### Self-reported physical activity

The Neighborhood Physical Activity Questionnaire (NPAQ) [44] captured usual weekly minutes of walking for transport (WT) and leisure (WL) inside and outside the neighborhood (within a 10–15 minute walk from home), as well as minutes of other recreational moderate-intensity (MPA) and vigorous-intensity (VPA). The NPAQ has acceptable test-retest reliability [44,45] and is associated with neighborhood built characteristics in the Canadian context [46,47]. Physical activity was captured at baseline, 4-week follow-up, and 12-week follow-up. Pedometer data were not available prior to participants registering in UWALK thus, we do not have a true pre-intervention baseline measure of steps and do not present these data.

#### Self-reported screen-based leisure time

Participants were asked “On average how many hours per week do you spend watching television or using a computer, tablet, iPad, smartphone outside of work time? (e.g., video-games, computer games, DVD/movies, internet, email, texting, etc.)”. A similar item has been used previously to measure leisure-based screen time in Canadian adults and was found to be associated with the built environment [36,48]. The item used in our study was updated to capture contemporary sedentary behaviors (e.g., mobile technology) not included in the original item [36]. Leisure-based screen time was captured at baseline, 4-week follow-up, and 12-week follow-up.

#### Perceived motivation

Self-efficacy reflects a person’s belief that they can successfully perform a behavior even when faced with barriers and obstacles that would otherwise inhibit or make performing the behavior difficult [49]. To capture self-efficacy in relation to accumulating steps during UWALK, at baseline participants responded to the three items, each having 7-point response options: 1) “How much control do you have over whether you achieve 10,000 steps on most days during the next month (no control to complete control)”; 2) “For me, achieving 10,000 steps on most days during the next month will be (very difficult to very easy)”, and; 3) “I am confident that I can achieve 10,000 steps on most days during the next month (not at all confident to completely confident)”. Item responses were averaged, with higher scores representing higher self-efficacy (internal consistency: Cronbach’s α = 0.74).

Similarly, to capture baseline intentions in relation to accumulating steps during UWALK, participants responded to three statements (with 7-point response options): 1) “I intend to walk 10,000 steps on most days during the next month (definitely do not to definitely do); 2) “I will make an effort to walk 10,000 steps on most days during the next month (definitely false to definitely true)”, and; 3) I will try to walk 10,000 steps on most days during the next month (definitely will not to definitely will). Item responses were averaged with higher scores indicating stronger intentions (internal consistency: Cronbach’s α = 0.94).

#### Perceived neighborhood walkability

At baseline, the Neighborhood Environment Walkability Scale (NEWS-A) [50] captured participant’s perceptions of built characteristics within a 15-minute walk from home (i.e., residential density, street connectivity, land use mix, park and transit proximity, aesthetics, traffic safety and safety from crime). Responses to 24-items were measured on a 4-point scale (i.e., strongly disagree to strongly agree). NEWS-A items have acceptable reliability and validity [51] and are associated with physical activity among Canadian adults [52]. Similar to a previous study [53], we recoded item responses into disagree (0) and agree (1) and created an index that reflected the sum of supportive walkability characteristics in the neighborhood (internal consistency: Cronbach’s α = 0.80).

#### Perceived neighborhood preferences

Preferences for particular neighborhood built characteristics may reflect lifestyle decisions, including physical activity, and residential choices [54]. During the baseline telephone survey, on a 5-point bipolar response scale (i.e., strongly agree to strongly disagree), participants reported their preference to: 1) “Live within walking distance to shops, restaurants, and other amenities”; 2) “Live within walking distance to public transit”; 3) “Live within walking distance to parks”; 4) “Live within walking distance to recreational facilities”, and; 5) “Live in a neighborhood where there are sidewalks and walking paths”. Item responses were averaged, with lower index scores representing a preference to reside in a neighborhood that was supportive of walking (internal consistency: Cronbach’s α = 0.65).

#### Sociodemographic characteristics (covariates)

During the baseline survey participants reported their age, sex, self-rated health (scored 1 = poor to 5 = excellent), highest education achieved (high school or less, college/vocation/trade, or university), annual gross household income (≤$80000, $80,000–119,000, ≥$120,000, or unknown/refused to answer), number of dependents <6 years of age and dependent 6–18 years of age at home, dog ownership (owner, non-owner), marital status (married/common law or other) and availability of a motor vehicle for personal use (always/sometimes, never/do not drive).

### Statistical analysis

**Fig 1:**
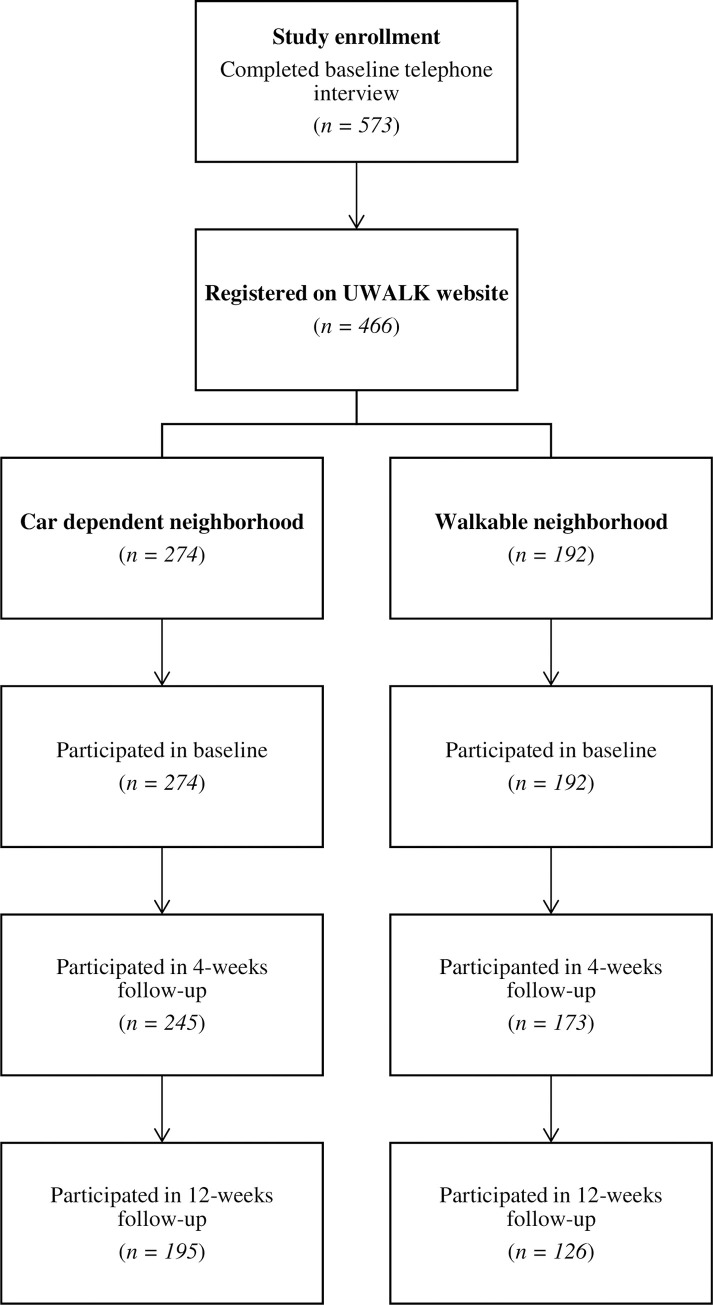
Study participation by neighborhood type.

Using Pearson’s chi-square and independent t tests, baseline sociodemographic and physical activity related characteristics were compared between those who registered (n = 466) and did not register (n = 107) for UWALK, between study completers (n = 321) and non-completers (n = 145), and between those residing in car dependent (n = 274) and walkable (n = 192) neighborhoods (Fig 1).

Linear mixed models (restricted maximum likelihood) were used to estimate the differences in physical activity and leisure screen activity between the car dependent and walkable neighborhood groups over three time periods (baseline, 4-week follow-up, and 12-week follow-up). A main effect for group (two levels: car dependent and walkable), a main effect for time (three levels: baseline, 4-weeks, and 12-weeks], an interaction term (group × time), and main effects for all covariates were included as fixed factors in the models. An unstructured covariance matrix was specified (random intercept and a random slope) to allow baseline outcomes and their slopes over time to vary between individuals. Within-group (car dependent vs. walkable) and between-group (by time) effects were analyzed using pairwise comparisons of the estimated marginal means derived from the models. Note, for the between group (time) effect, the 4-week and 12-week follow-ups were each compared with baseline (reference category). The models were adjusted for all baseline sociodemographic covariates (i.e., gender, age, education, income, marital status, children, dog ownership, motor vehicle access, self-efficacy, intention, neighborhood preferences, and perceived walkability) and 95% confidence intervals (CI) estimated. The average number of participants clustering per postal code was 1.07 (SD 0.30), thus we did not adjust for clustering in our analysis [55]. Analysis was undertaken using Stata 15 (Stata Corp LLC, Texas, USA).

## Results

### Study enrollment and participation

Among the participants who registered for UWALK, the majority were female (83%), had a university education (60.7%), were married or common-law (69.3%), did not own a dog (79%), and had access to a motor vehicle always or sometimes (91.2%) (Table 1). Almost one-third (31.5%) of participants reported a household income of <$80,000/year. On average participants were 49.2 years (SD = 14.4), had 0.3 (SD = 0.6) children under 6 years of age and 0.4 (0.9) children 6–18 years of age at home. Participants were also in good or better health (mean = 3.0; SD = 0.9], perceived their neighborhood to be highly walkable (mean = 18.7; SD = 3.4), preferred to live in neighborhoods with characteristics that supported walking (mean = 1.4; SD = 1.4), and had high intentions (mean = 6.0; SD = 1.3) and self-efficacy (mean = 5.0; SD = 1.3) in relation to UWALK (Table 1). Compared with eligible participants who did not register for UWALK, those who registered included a significantly (p < .05) higher proportion of participants who were married/common-law and had higher self-reported health. Baseline weekly MPA was also significantly (p < .05) higher among those who registered versus those who did not register for UWALK (mean = 37.9; SD = 77.7 vs. mean = 22.6; SD = 40.4 minutes/week, respectively). Apart from dog ownership (p < .05), participants who completed all three surveys (completers) were not significantly different to those who did not complete all three surveys (non-completers) on all baseline characteristics (including physical activity variables) (Table 1). Notably, walkability (i.e., Walk Score®) was not significantly different between those registered versus not registered for UWALK nor between completers versus non-completers.

**Table 1:** Study enrollment, registration for UWALK, and study completion by baseline characteristics (T1).

		Study Enrollment		Study Completion	
		Registered for UWALK (n = 466)	Did not registered for UWALK (n = 107)	Completer (n = 321)	Non-completer (n = 145)
		Estimate [% or Mean(SD)]	Estimate [% or Mean(SD)]		
Age in years		49.2 (14.4)	50.1 (14.6)	49.9 (14.3)	47.4 (14.5)
Sex	Female	83.0	77.6	81.9	85.5
	Male	17.0	22.4	18.1	14.5
Highest education	High school or less	15.0	17.8	14.3	16.5
	College or trade	24.3	23.3	25.2	22.1
	University	60.7	58.9	60.5	61.4
Marital status	Married/common-law	69.3^a^	58.9	69.5	69.0
	Other	30.1^a^	41.2	30.5	31.0
Annual household income	Less than $80,000	31.5^a^	46.7	30.2	34.5
	$80,000 to 119,999	16.3^a^	6.5	18.1	12.4
	$120,00 or more	15.9	17.8	14.3	19.3
	Unknown	36.3	29.0	37.4	33.8
Children < 6 years at home		0.3 (0.6)	0.2 (0.5)	0.3 (0.6)	0.2 (0.5)
Children 6–18 years at home		0.4 (0.9)	0.6 (1.0)	0.4 (0.9)	0.5 (0.9)
Dog at home	No dog	79.0	83.2	81.8^b^	72.4
	Non owner	21.0	79.8	18.1^b^	27.6
Motor vehicle access	Always or sometimes	91.2	94.4	91.3	91.0
	Never or don’t drive	8.8	5.6	8.7	9.0
Self-rated physical health		3.0 (0.9)^a^	2.8 (1.0)	3.0 (0.8)	2.9 (1.0)
Walk Score®		44.7 (21.3)	44.3 (19.5)	43.9 (21.2)	46. 3 (21.5)
Perceived walkability		18.7 (3.4)	18.3 (3.4)	18.6 (3.4)	19.0 (3.3)
Preference for walkability		1.4 (0.5)	1.4 (0.5)	1.4 (0.5)	1.4 (0.5)
Intention (10,000 steps/day)		6.0 (1.3)	5.9 (1.3)	5.9 (1.3)	6.1 (1.2)
Self-efficacy (10,000 steps/day)		5.0 (1.3)	4.9 (1.3)	4.9 (1.3)	5.1 (1.2)

^a^p < .05 for difference between enrollment in study.

^b^p < .05 for difference between study completion.

Pearson Chi-Square for frequencies and Independent t-test for continuous variables.

“Completer”: Participation in baseline, 4 week follow-up, and 12 week follow-up surveys.

“Non-completer”: Participated in baseline, but did not participate in all follow-up surveys.

### Sociodemographic characteristics by neighborhood walkability

No significant differences existed in the proportion of participants from car dependent and walkable neighborhood groups who did not complete the 4-week (11% vs. 10%) and 12-week (29% vs. 34%) follow-up surveys (Table 1). However, compared with participants from car dependent neighborhoods, those from walkable neighborhoods had a significantly higher proportion of households incomes with less than $80,000/year (37.5% vs. 27.4%), fewer children 6–18 years of age (mean = 0.3 vs. 0.6 per household), and higher proportion reporting not driving or never having access to a motor vehicle for personal use (13.5% vs. 5.5%) (Table 2).

**Table 2:** Baseline characteristics by neighborhood type among those who registered for UWALK.

		Neighborhood Type	
		Car Dependent (n = 274)	Walkable (n = 192)
		Estimate [% or Mean(SD)]	Estimate [% or Mean(SD)]
Age in years		49.1 (14.2)	49.2 (14.7)
Sex	Female	82.1	84.4
	Male	17.9	15.6
Highest education	High school or less	14.2	16.1
	College or trade	24.1	24.5
	University	61.7	59.4
Marital status	Married/common-law	71.9	65.6
	Other	28.1	34.4
Annual household income	Less than $80,000	27.4^a^	37.5
	$80,000 to 119,999	17.1	15.1
	$120,00 or more	17.5	13.5
	Unknown	38.0	33.9
Children < 6 years at home		0.3 (0.5)	0.3 (0.6)
Children 6–18 years at home		0.5 (1.0)^b^	0.3 (0.7)
Dog at home	No dog	77.4	81.2
	Non owner	22.6	18.8
Motor vehicle access	Always or sometimes	94.5^b^	86.5
	Never or don’t drive	5.5^b^	13.5
Self-rated physical health		2.9 (0.9)	3.1 (0.8)
Walk Score®		29.9 (11.6)^c^	65.7 (12.4)
Perceived walkability		18.5 (3.1)	19.0 (3.7)
Preference for walkability		1.5 (0.5)	1.4 (0.5)
Intention (10,000 steps/day)		6.0 (1.1)	5.8 (1.4)
Self-efficacy (10,000 steps/day)		5.0 (1.2)	4.9 (1.4)

^a^ p < .05

^b^ p < .01

^c^ p < .001 for difference between neighborhood type (Pearson Chi-Square for frequencies and Independent t-test for continuous variables).

### Differences in physical activity by neighborhood walkability during UWALK

Adjusting for all covariates, weekly minutes of WT inside the neighborhood was significantly (p < .001) higher among participants from walkable versus car dependent neighborhoods at baseline (42.5; CI = 35.9, 49.2 vs. 21.1; CI = 15.5, 26.6), 4-week follow-up (81.2; CI = 67.4, 95.1 vs. 48.2; CI = 36.6, 59.8), and 12-week follow-up (87.2; CI = 70.4, 104.1 vs. 48.0; CI = 34.3, 61.6) (Table 3). Despite no significant difference at baseline, compared with participants from car dependent neighborhoods, those from walkable neighborhoods also reported significantly (p < .05) more weekly minutes of WT outside the neighborhood (68.6, CI = 52.9; 84.4 vs. 47.1; CI = 33.9, 60.3) and more WL inside the neighborhood (161.0; CI = 135.8, 186.3 vs. 126.7; CI = 105.5, 147.9) at the 4-week follow-up. No other neighborhood group within-time differences were found.

**Table 3:** Estimated adjusted means and difference between neighborhood type and time during UWALK.

	Adjusted Means (CI)			Adjusted Differences (CI)		p-value (Group by Time Interaction)
	Baseline	4-weeks	12-weeks	Baseline vs. 4-weeks	Baseline vs. 12-weeks	
**WT in neighborhood (min/week)**						
Car dependent	21.1 (15.5, 26.6)	48.2 (36.6, 59.8)	48.0 (34.3, 61.6)	**27.1** **(15.9, 38.2)** ^c^	**26.9** **(13.1, 40.7)** ^c^	0.222
Walkable	**42.5** **(35.9, 49.2)** ^c^	**81.2** **(67.4, 95.1)** ^c^	**87.2** **(70.4, 104.1)** ^c^	**38.7** **(25.3, 52.0)** ^c^	**44.7** **(27.7, 61.7)** ^c^	
					
**WT out neighborhood (min/week)**						
Car dependent	25.9 (16.8, 35.0)	47.1 (33.9, 60.3)	60.7 (42.9, 78.6)	**21.2** **(6.2, 36.3)** ^b^	**34.9** **(6.0, 53.7)** ^c^	0.059
Walkable	24.2 (13.3, 35.1)	**68.6** **(52.9, 84.4)** ^a^	61.2 (39.3, 83.1)	**44.5** **(26.5, 62.4)** ^c^	**37.0** **(13.9, 60.1)** ^b^	
					
**WL in neighborhood (min/week)**						
Car dependent	40.0 (31.4, 48.7)	126.7 (105.5, 147.9)	145.9 (119.2, 172.5)	**86.6** **(66.3, 107.0)** ^c^	**105.8** **(78.5, 133.1)** ^c^	0.216
Walkable	46.5 (36.1, 56.9)	**161.0** **(135.8, 186.3)** ^a^	173.4 (140.4, 206.3)	**114.5** **(90.3, 138.8)** ^c^	**126.8** **(93.2, 160.5)** ^c^	
					
**WL out neighborhood (min/week)**						
Car dependent	25.0 (18.1, 32.0)	94.8 (77.3, 112.4)	104.3 (82.6, 126.1)	**69.8** **(52.3, 87.3)** ^c^	**79.3** **(57.2, 101.3)** ^c^	0.661
Walkable	31.3 (23.6, 40.1)	91.3 (70.4, 112.2)	97.0 (70.1, 123.9)	**59.4** **(38.6, 80.3)** ^c^	**65.2** **(38.0, 92.4)** ^c^	
					
**LMPA (min/week)**						
Car dependent	36.8 (27.6, 46.2)	120.4 (100.0, 140.9)	141.1 (112.6, 169.5)	**83.6** **(62.0, 105.2)** ^c^	**104.2** **(74.4, 134.0)** ^c^	0.781
Walkable	39.2 (28.1, 50.3)	111.8 (87.5, 136.2)	133.6 (98.2, 168.9)	**72.6** **(46.9, 98.4)** ^c^	**94.3** **(57.4, 131.3)** ^c^	
					
**LVPA (min/week)**						
Car dependent	21.1 (16.4, 25.7)	63.6 (45.8, 81.4)	68.7 943.6, 93.8)	**42.5** **(24.6, 60.5)** ^c^	**47.6** **(22.2, 73.1)** ^c^	0.306
Walkable	21.7 (16.1, 27.3)	60.3 (39.1, 81.6)	96.3 (65.3, 127.3)	**38.7** **(17.3, 60.0)** ^c^	**74. 7** **(5.0, 66.9)** ^c^	
					
**Leisure screen activity (hr/week)**						
Car dependent	20.3 (18.9, 21.8)	16.0 (14.5, 17.6)	14.8 (13.2, 16.3)	**-4.3** **(-5.9, -2.8)** ^c^	**-5.6** **(-7.2, -3.9)** ^c^	0.507
Walkable	20.6 (18.9, 22.3)	17.5 (15.7, 19.4)	16.4 (14.5, 18.3)	**-3.1** **(-4.9, -1.2)** ^c^	**-4.2** **(-6.2, -2.2)** ^c^	
					
					

Adjusted means and differences estimated with all covariates (gender, age, education, income, marital status, children, dog ownership, motor vehicle access, self-efficacy, intention, neighborhood preference, and perceived walkability) and group by time interaction included in the linear mixed model.

Pairwise comparisons between groups within time compared to “car dependent” neighborhoods. Pairwise comparisons within group between time points compared to baseline. CI: 95% confidence interval. WT: Walking for transportation. WL: Walking for leisure. LMPA: Leisure-time moderate-intensity physical activity (excluding walking). VPA: Leisure-time vigorous-intensity physical activity.

^a^ p < .05

^b^ p < .01

^c^ p < .001.

The within group by time differences showed that all physical activity outcomes were higher at 4-weeks and 12-weeks compared with baseline among participants from car dependent and walkable neighborhoods (Table 3). Similarly, compared with baseline, leisure screen time was lower at the 4-week and 12-week follow-up for both groups. Notably, much of the observed change in physical activity and leisure screen time occurred within the 4-weeks after baseline. We found no significant group by time interactions suggesting that during the 12-week intervention, UWALK had similar effects on all physical activity outcomes and leisure-based screen time for participants from car dependent and walkable neighborhoods (Table 3).

## Discussion

We hypothesized that changes in self-reported physical activity at 4 weeks and 12 weeks during the intervention would be significantly higher for adults residing in neighborhoods that were more versus less walkable. Adding to the mixed evidence [32] and contrary to our hypothesis, we found that level of neighborhood walkability did not affect changes in physical activity and sedentary behavior resulting from participation in an individual-level physical activity intervention. Congruent with previous studies [3,5,6] we did, however, find increases in physical activity as a result of participating in a short-term internet-delivered pedometer-facilitated intervention, independent of neighborhood walkability. Our findings extend this previous evidence by demonstrating that pedometer-facilitated interventions can support changes in different types and intensities of physical activity and physical activity undertaken for different purposes (i.e., leisure vs. transportation) regardless of levels of neighborhood walkability. Our finding that participation in a 12-week pedometer intervention is associated with a reduction in leisure-based screen activity also aligns with previous findings related to sedentary behavior [56].

In our study, we did not find any statistically significant group (car dependent vs. walkable) by time (baseline, 4-week, vs. 12-week) interactions, suggesting that UWALK had a similar effect on changes in time spent on physical activity and leisure-based screen activity regardless of neighborhood walkability. At the conclusion of the 12-week intervention, participants from car dependent and walkable neighborhoods similarly had approximately doubled or in some cases tripled their weekly minutes of transport and leisure walking inside and outside their neighborhood, and leisure-based moderate and vigorous intensity physical activity. The greatest relative improvements in physical activity were observed within the first 4-weeks of the intervention, which tended to be maintained until week 12. These findings are encouraging as they suggest internet-delivered pedometer-facilitated interventions can influence physical activities in addition to walking, and such improvements in physical activity could provide health benefits [2].

Contrary to our findings, other studies have found that the neighborhood built environment can affect the physical activity response to individual-level interventions [32], including those that use pedometers [33–35]. Our findings are based on a sample of adults who were in the contemplation and preparation stages of behavior change, and included mostly of women and those highly educated. Moreover, our sample on average had relatively high self-efficacy and intention, and positive perceptions of neighborhood walkability. In particular, high levels of self-efficacy may have allowed participants to persevere when they encountered obstacles, such as built environment features unsupportive of physical activity, during the UWALK intervention [49]. In our previous study, which included perceived overall walkability and Walk Score®, only the former was positively associated with the accumulation of steps during the same 12-week intervention [38]. Compared with objectively measured walkability, perceptions of walkability might be more strongly linked with changes in physical activity associated with pedometer-facilitated interventions. Individuals who perceive their neighborhoods to be less walkable may require additional support (e.g., receiving detailed neighborhood maps showing convenient, safe, and attractive walking routes and amenities) [57,58] to encourage them to accrue the full benefits from participating in pedometer interventions. Our findings overall show that the UWALK intervention appears to be effective for encouraging immediate and short-term improvements in walking, and moderate-intensity and vigorous-intensity physical activity regardless of neighborhood walkability suggesting that the intervention could be implemented more broadly in different neighborhood contexts.

We found that weekly minutes of neighborhood transportation walking at baseline was higher among participants from walkable versus car dependent neighborhoods. The difference between the walkable and car dependent neighborhoods remained at the 4-week and 12-week follow-up, despite participants in both groups increasing their neighborhood transport walking minutes. The positive association of neighborhood walkability with transport walking is consistent with previous findings [18,29,59]. Improving neighborhood walkability is important for supporting physical activity, in particular transport walking. However, our findings also suggest that pedometer-facilitated interventions might encourage other types of physical activity regardless of neighborhood walkability. Our findings may not extend to other types of physical activity interventions (e.g., motivational interviewing, mass media campaigns). For instance, in an Australian study, objectively-measured neighborhood walkability did not modify the effects of a mass media campaign (*Find Thirty every day®*) on physical activity (i.e., transport walking, overall walking, and total physical activity) [60], while in a US study, the effects of a media campaign (*Wheeling Walks*) on changes in walking were moderated only by the positive perceptions related to neighborhood aesthetics and facilities [61].

A notable finding of our study was the positive effect of UWALK on leisure-based screen activity. In their meta-analysis, Qiu et al. [56] found that pedometer-facilitated interventions (8 to 48 weeks in duration) reduced non-specific sedentary time by approximately 23 minutes per day (or 2.7 hours/week). Our study did not include total sedentary time, nevertheless we found that participation in UWALK was associated with a reduction in leisure-based screen time of about 3–4 hours per week at 4-weeks and 4 to 5 hours per week at 12 weeks. These findings suggest that pedometer-facilitated interventions encourage physical activity while also reducing sedentary time; both of which are independent risk factors for chronic disease [1,62,63]. Importantly, our findings extend this knowledge by demonstrating that neighborhood walkability may not moderate the effect of pedometer-facilitated interventions on leisure-based screen time.

Despite the null findings, to our knowledge this is the first study to estimate the effect of an interaction between objectively-measured walkability and a physical activity intervention on context (i.e., neighborhood) specific physical activity [32]. Our study has several positive attributes such as measuring change in a range of context-specific physical activities over three time points, statistical adjustment for baseline motivational characteristics (intention, self-efficacy), perceived walkability, preference for residing in a walkable neighborhood, self-rated health, and sociodemographic characteristics, using an established walkability index (Walk Score®), and recruitment of adults who were in the contemplation and preparation stages of physical activity change. Our study also has several limitations. Despite using equivalent questions, the baseline survey involved a telephone-interview while the 4-week and 12-week surveys were self-administered using an online questionnaire. We cannot dismiss the effect of survey modality on the observed behavior changes between baseline and follow-up [64], although this effect is likely to be small [65]. We also cannot rule-out systemic external factors that may have contributed to behavior change; however, the magnitude of changes observed during the 12-week intervention together with supporting qualitative findings [37] suggest that UWALK likely influenced physical activity and sedentary behavior. Nevertheless, the inclusion of a control group along with randomization would have strengthened the study’s internal validity. Self-reported physical activity and sedentary behavior are susceptible to recall and memory bias [66,67] and despite having pedometer data, we did not capture pre-intervention steps. While Walk Score® is correlated with other objective walkability indices [24–26], built features that are not captured by Walk Score® could have impacted individual responses to UWALK. Individual responses to UWALK might also differ when more extreme Walk Score® classifications are compared (e.g., very car-dependent vs. walker’s paradise) [28,29] however, our sample size and the distribution of walkability scores did not provide sufficient statistical power to explore more refined group differences. Finally, participants self-selected into the study and the potential volunteer bias limits the external validity of our findings.

## Conclusions

Pedometer-facilitated interventions have the potential to provide immediate positive impacts on different physical activities and sedentary behavior. Importantly, individuals residing in low walkable or car-dependent neighborhoods participating in pedometer-facilitated interventions similarly benefit in terms of improved physical activity and sedentary behavior compared with those residing in walkable neighborhoods. However, more research is needed to explore the effect of objectively-measured and perceived neighborhood built features on the physical activity and sedentary behavior impacts of different types of interventions.
